# The Effects of Intraarticular Opioids in pain relief after Arthroscopic Menisectomy: A Randomized Clinical Trial Study

**DOI:** 10.12669/pjms.292.2809

**Published:** 2013-04

**Authors:** Hamidreza Arti, Sara Arti

**Affiliations:** 1 Hamidreza Arti, Associate Professor of Orthopaedic Surgery, Ahvaz Jundishapur University of Medical Sciences, Ahvaz, Iran.; 2 Sara Arti, Ms. in Midwifery and Biostatistics, Vice Chancellery for Research, Isfahan University of Medical Sciences, Isfahan, Iran.

**Keywords:** Arthroscopy, Morphine, Meperidine, Methadone, Intraarticular Analgesia

## Abstract

***Objective: ***Knee arthroscopy is a painful procedure which if untreated will cause intensive and prolonged pain that may prevent rehabilitation of patients. This study was designed to compare the analgesic effects of different opioids in the early post operative period in comparison to control group.

***Methodology:*** One hundred forty patients were prospectively assigned to four groups randomly. After arthroscopic menisectomy all patients received an intraarticular injection containing 9.5 ml bupivacaine 0.5% with 1:200000 epinephrine in a 10 ml syringe. The remainder of syringe was filled with one of the study solutions. Group I: 5mg methadone, group II: 5mg morphine, group III: 5 ml normal saline, group IV: 50 mg meperidine. At three stages in the ealy post operative period the need for analgesics was recorded. A statistical comparison was done afterwards.

***Results*** *:* In morphine group (group II), the analgesic usage in hospitalized and outpatients compared with other groups was significantly low(P<0.05).

***Conclusion:*** Morphine in comparison to meperidine or methadone is more beneficial in reducing pain or analgesic need when is added to bupivacain injection following arthroscopic menisectomy.

## INTRODUCTION

Arthroscopic menisectomy is a common procedure which is accompanied by post operative pain that is the most important factor in preventing patient rehabilitation.^1^ In this procedure usually intraarticular injections of local anesthetics are used but nowadays multimodal analgesia as combined methods are recommended so as to apply synergistic effects of drugs to minimize the administered dosage.^2^ The effects of pain relief of intraarticular narcotic analgesic drugs following arthroscopy menisectomy or anterior cruciate ligament reconstruction have been shown.^3^^,^^4^ Morphine, methadone and meperidine are the narcotic analgesics that have been evaluated for this purpose.^5^^-^^7^ The intraarticular dosage of morphine vary in different studies.^8^^,^^9^ Methadone is a potent and long acting drug that has a high affinity and activity on opioid receptors. It bonds strongly to proteins and has a low solubility in lipids with a half life of about 35 hours.^10^^,^^11^Meperidin also is an opioid analgesic with a half life of four hours and its analgesic effects lasts 12-16 hours after intra-articular injection.^10^^,^^11^

 Analgesic effects of different opioids have been compared in various studies but according to our study, no survey has been made on the comparison of intra-articular injection of morphine, meperidine and methadone in arthroscopic menisectomy in different large groups.

 In this study, our purpose was the comparison of the effects of intraarticular injection of morphine, meperidine, methadone and normal saline (control group) in combination with Bupivacaine in pain relief after arthroscopic menisectomy.

## METHODOLOGY

In a prospective, double blind, randomized clinical trial study, 178 patients with meniscal tears who were referred to Ayatollah Kashani Hospital of Shahrekord between years 2005-2010 were evaluated. Thirty eight patients were excluded because of having multiple injuries in knee or their reluctance to continue participation in this study or applying tourniquets during operation. The 140 remaining patients were informed about the method of analgesia following the procedure and they signed a written informed consent.

***The inclusion criteria were:*** 1. Ages between 18-65 years 2. Class I or II health status according to American society of Anesthesia (ASA). 3. Possibility of doing the procedure by general anesthesia.

***The exclusion criteria were: ***1. History of sensitivity to any of the drugs under study. 2. Addiction to opioids. 3. History of chronic pain. 4. Pregnancy or breast feeding. 5. Using drains following operation. 6. Consumption of MAOI drugs.^11^ 7. Preoperative consumption of opioid drugs or other analgesics. 8. Using tourniquet during operation. 9. Diseases or problems that may influence the results.

All the patients underwent general anesthesia by a similar standard anesthetic protocol. Tourniquet was not used during operation. Before operation, patients randomly enrolled into one of four groups (with 35 patients) and had intra-articular injection of 10 ml of one of 4 solutions respectively:

Group I: Bupivacain 0.5% plus Epinephrine 1:200,000 (9.5 ml) and 5 mg of methadone (0.5 ml).

Group II: Bupivacain 0.5% plus Epinephrine 1:200,000 (9.5 ml) and 5 mg of morphine (0.5ml).

Group III (control group): Bupivacain 0.5% plus Epinephrine 1:200,000 (9.5 ml) and normal saline (0.5 ml).

Group IV: Bupivacain 0.5% plus Epinephrine 1:200,000 (9.5 ml) and 50 mg meperidine (0.5 ml).

Randomization of administered drugs was done in the pharmacy based on randomization number tables. Sterile syringe content was injected from superolateral area of knee after arthroscopy and no drain or other evacuation system was use. The substances in the injecting syringes were unknown to surgeons, Anesthesiologists and the patients. A list of syringe numbers and content drugs was prepared in the pharmacy for final evaluation. The drugs for pain relief after operation were intravenous morphine and acetaminophen codeine tablets that were used for all patients according to the patients need during hospitalization. The dosage and the method of administration was registered and the morphine equivalent dosage was calculated according to the potency of 10 mg of morphine sulfate which is equal to 10 mg methadone hydrochloride, 100 mg of meperidine hydrochloride, 200 mg of codeine or 600 mg of acetaminophen plus 60 mg of codeine.^11^^,^^12^ The visual analogue scale were registered according to each analgesic request every 4 hours. All patients were discharged with prescription of Acetaminophen codeine tablets for pain relief the day after operation and asked for registering the time and amount of analgesic consumption after discharge and the data were obtained until 7 days following operation.

The data were gathered by checklists questionnaires and then were statistically analyzed by SPSS_18_. The random data were analyzed by Chi-square test. The need for analgesics after operation scores on visual analogue scale were analyzed by analysis of variance. If analysis of variance showed any significant difference between groups, the Tukey test was used to show the better differences. Seven mg difference in morphine equivalent and 8 mg standard deviation between groups^13^ and also (p< 0.05) was considered meaningful and significant.

## RESULTS

Over all 140 patients were enrolled in the study from which, 35 were given intraarticular methadone, 35 morphine, 35 normal saline and 35 ones meperidine. No demographic differences among age, height sex, and weight were found in groups. Analysis of analgesic combinations converting to morphine equivalent showed significant difference between groups (Table-I). In the first step of the study that was the period between anesthesia and complete recovery of patient and the effect of anesthetics were fading out, the Tukey test showed lower pain standard in morphine group in contrast to methadone, meperidine or control group (p< 0.05).

The average analgesic request in morphine group was less than other groups (p< 0.05). In this instance, comparison with groups by Tukey test showed no difference between methadone or meperidine injected groups and control group (p> 0.05).

In the second stage of the study that was from recovery to discharge on the first post-operation day, there was significant less pain in the morphine injected group in comparison with methadone, meperidine and control group (p< 0.05).

In the third stage in the outpatient care period from discharge till 7 days post operation, the analysis of the average analgesic request showed significant difference between groups, it was less in morphine group than other groups (p< 0.05) (Table-I).The analysis of data by using Tukey test showed no significant difference between methadone group and control group (p> 0.05) or meperidine group and control group (p> 0.05).

**Table-I:** The average analgesic request and pain scores in patients underwent knee arthroscopic menisectomy

Group	*Number of* *Patients*	*Visual analogue scale* *following end of anesthesia till complete recovery*	*Analgesic request in hospitalized patients* *Mean ± SD*	*Analgesic request in outpatient groups from discharge till 7 days postoperation* *Mean ± SD*
I (Methadone )	35	3.87 ± 3.3	22 ± 15.3	35 ± 15.1
II( Morphine )	35	2.02 ± 3.4	10.5 ± 9.4	23.5 ± 16
III (Normal saline)	35	5.3 ± 3.3	21.4 ± 13.3	38.1 ± 19.6
IV (Meperidine)	35	3.40 ± 2.3	23 ± 17.2	34 ± 14.3

The need for analgesia during hospitalization (p< 0.05) and in outpatients (p< 0.05) and also pain score in morphine group patients (group II) was less in comparison to other groups but there was no significant difference between group I, II, and III.

Moreover, there was no significant difference between Methadone and control groups or between Methadone and Meperidine groups (p< 0.05). There was no significant difference in pain score in hospitalized or out patients in other times (p>0.05) and no side effect due to intra-articular injection was detected.

## DISCUSSION

 In the present study, intra-articular Morphine administration had a more powerful analgesic affect in comparison with control group. Similar results were reported by using different doses of intraarticular morphine injection.^5^^,^^6^ There was less analgesic request in 24 hours post operation in the group II (morphine group). Intra-articular injection of morphine in comparison with intravenous injection gives better analgesic effects and provides lower analgesic needs in patients.^14^

Intraarticular injection of morphine has prolonged analgesic effects for 8-12 hours. The presence of morphine receptors in peripheral nerve branches has been proven. Two mechanisms responsible for analgesic effects of local morphine administration are: removing of neural sheath in neural terminals so that it permits access of morphine substance to nerve receptors existence of inflammation which causes activation of inactive morphine receptors, increases local tissue bonds to morphine substance and both of these mechanisms takes place during arthroscopic menisectomy.^7^

Direct injection of morphine to the inflamed tissue of meniscus or synovium leads to increased duration and potency of its effect.^4^ Ten minutes after inflating the tourniquets, morphine substances cause intensive analgesic effects probably due to increased tissue bonds.^7^^,^^15^^,^^16^ That is why tourniquet was not used in this study. Another factor influencing the effectiveness of intraarticular injection of morphine is the drug dosage. High doses of morphine accompanies the effect on central nervous system receptors and intra-articular morphine injection affects on injured tissues caused decreasing drug need, better analgesia and reducing side effects.^15^

A considerably unexpected results in our study was that methadone and meperidine do not have significant analgesic effect, because these two drugs produce their analgesic effects through stimulation opioid receptors.^10^^,^^16^

One of the reasons of nonfunctioning of the other drugs except morphine in our study is probably due to their pharmacokinetic differences.^2^^,^^10^^,^^11^ May be morphine has the ability to reach peripheral opioid receptors but methadone and meperidine do not have adequate power. The effectiveness of 5 mg intraarticular morphine injection following Anterior cruciate ligament reconstruction and in meniscectomy had been shown before as well as in this study.^5^^,^^16^^,^^17^ Intraarticular injection of 10 mg morphine is the maximum intraarticular dosage reported in the literature but it is not directly compared with the common dosage of 5 mg or more is not yet permitted but intra articular methadone injection of this dose of opioids is safe in human being.

Our study showed that intra-articular injection of 5 mg morphine is safe and useful in lowering the need for analgesia, but this effect is just significant in the first 24 hours which is similar to other studies. The fall in efficacy later may be due to subsiding inflammatory reaction afterwards.^18^^-^^20^ A combination drug regime including bupivacain, epinephrin and intraarticular analgesic if used will require small dose and will also have minimum side effects with maximum of probable synergistic quality.

## CONCLUSION

In summary it can be safely concluded from our results methadone or meperidine do not seem to have efficacy in analgesia following arthroscopic menisectomy whereas 5 mg of intra articular morphine injection is safe and useful for analgesia following arthroscopic meniscectomy. Using this drug in combination with bupivacain is recommended in arthroscopic meniscectomy.
